# Combined Effects of PFAS, Social, and Behavioral Factors on Liver Health

**DOI:** 10.3390/medsci13030099

**Published:** 2025-07-28

**Authors:** Akua Marfo, Emmanuel Obeng-Gyasi

**Affiliations:** 1 Department of Built Environment, North Carolina A&T State University, Greensboro, NC 27411, USA; 2 Environmental Health and Disease Laboratory, North Carolina A&T State University, Greensboro, NC 27411, USA

**Keywords:** PFAS, behavior, exposome, liver function, socioeconomic status, environmental health

## Abstract

Background: Environmental exposures, such as per- and polyfluoroalkyl substances (PFAS), in conjunction with social and behavioral factors, can significantly impact liver health. This research investigates the combined effects of PFAS (perfluorooctanoic acid (PFOA) and perfluorooctane sulfonate (PFOS), alcohol consumption, smoking, income, and education on liver function among the U.S. population, utilizing data from the 2017–2018 National Health and Nutrition Examination Survey (NHANES). Methods: PFAS concentrations in blood samples were analyzed using online solid-phase extraction combined with liquid chromatography–tandem mass spectrometry (LC-MS/MS), a highly sensitive and specific method for detecting levels of PFAS. Liver function was evaluated using biomarkers such as alanine aminotransferase (ALT), aspartate aminotransferase (AST), alkaline phosphatase (ALP), gamma-glutamyltransferase (GGT), total bilirubin, and the fatty liver index (FLI). Descriptive statistics and multivariable linear regression analyses were employed to assess the associations between exposures and liver outcomes. Bayesian Kernel Machine Regression (BKMR) was utilized to explore the nonlinear and interactive effects of these exposures. To determine the relative influence of each factor on liver health, Posterior Inclusion Probabilities (PIPs) were calculated. Results: Linear regression analyses indicated that income and education were inversely associated with several liver injury biomarkers, while alcohol use and smoking demonstrated stronger and more consistent associations. Bayesian Kernel Machine Regression (BKMR) further highlighted alcohol and smoking as the most influential predictors, particularly for GGT and total bilirubin, with posterior inclusion probabilities (PIPs) close to 1.0. In contrast, PFAS showed weaker associations. Regression coefficients were small and largely non-significant, and PIPs were comparatively lower across most liver outcomes. Notably, education had a higher PIP for ALT and GGT than PFAS, suggesting a more protective role in liver health. People with higher education levels tend to live healthier lifestyles, have better access to healthcare, and are generally more aware of health risks. These factors can all help reduce the risk of liver problems. Overall mixture effects demonstrated nonlinear trends, including U-shaped relationships for ALT and GGT, and inverse associations for AST, FLI, and ALP. Conclusion: These findings underscore the importance of considering both environmental and social–behavioral determinants in liver health. While PFAS exposures remain a long-term concern, modifiable lifestyle and structural factors, particularly alcohol, smoking, income, and education, exert more immediate and pronounced effects on hepatic biomarkers in the general population.

## 1. Introduction

Per- and polyfluoroalkyl substances (PFAS) are a large class of synthetic chemicals that have been used in a wide range of industrial and consumer products since the 1950s [1]. Due to their chemical stability and resistance to degradation, PFAS are often referred to as “forever chemicals” [2]. They are commonly found in items such as non-stick cookware, water-repellent clothing, firefighting foams, food packaging, and construction materials [3]. Because of their widespread use and environmental characteristics, PFAS have become a global concern, with human exposure occurring through air, water, food, and household products [4,5,6].

Numerous studies have linked PFAS exposure to liver damage, particularly through biomarkers such as alanine aminotransferase (ALT) and gamma-glutamyltransferase (GGT) [6]. However, much of this research has examined PFAS in isolation, without adequately accounting for other vital contributors to liver health, such as alcohol use, smoking, income level, and education. These social and behavioral factors are well-established determinants of liver disease. For example, alcohol and tobacco use are known to cause liver inflammation and fibrosis [7,8,9,10,11,12], while individuals with lower income and education levels often face barriers to healthcare, healthy foods, and health-related knowledge, all of which may impact liver function [13,14,15].

Despite their significance, few studies have explored how these social and behavioral factors interact with environmental exposures like PFAS to influence liver health. Moreover, most existing research relies on traditional linear models, which may not fully capture the complex, nonlinear, and interactive nature of multiple exposures. This leaves a gap in our understanding of how real-world mixtures, both chemical and non-chemical, affect liver outcomes.

To address this gap, this study investigates the combined effects of PFAS, behavioral factors (alcohol use and smoking), and social factors (income and education) on liver function. We use nationally representative data from the 2017–2018 National Health and Nutrition Examination Survey (NHANES) and apply Bayesian Kernel Machine Regression (BKMR) to model the nonlinear and interactive relationships between these exposures and liver biomarkers, including ALT, AST, ALP, GGT, total bilirubin, and the fatty liver index (FLI).

To our knowledge, this is one of the first studies to apply BKMR to assess how PFAS exposures interact with behavioral and socioeconomic factors in relation to liver health. Our findings aim to provide a more comprehensive understanding of how lifestyle, social context, and environmental chemicals together shape liver health in the U.S. population.

## 2. Materials and Methods

### 2.1. Study Design

This investigation utilized data from the 2017–2018 cycle of the NHANES [16]. This is a program conducted by the Centers for Disease Control and Prevention (CDC) to assess the health and nutritional status of adults and children throughout the United States. NHANES combines in-depth interviews encompassing demographic, socioeconomic, dietary, and health-related variables with thorough physical examinations, laboratory tests, and clinical evaluations performed by qualified healthcare personnel. These data provide a crucial foundation for estimating disease prevalence, tracking health and nutritional trends, establishing national reference standards, and informing public health strategies and policy formulation. The survey encompasses a broad spectrum of chronic conditions and risk factors, including smoking, alcohol consumption, obesity, aging, and dietary habits, alongside diseases and outcomes such as cardiovascular disease, diabetes, respiratory conditions, and elements of reproductive health.

#### 2.1.1. Description of Cohort

The NHANES 2017–2018 dataset provides data for a total of 9254 participants, with 4557 males (49.2%) and 4697 females (50.8%). The study included participants aged 12 years and older to ensure a representative sample of both adolescents and adults, as liver health and the effects of PFAS are most relevant in this age group.

Participants were included in the analysis if they had complete data for serum PFAS measurements (PFOA and PFOS) and liver function biomarkers, including AST, ALT, ALP, GGT, and total bilirubin. As per the NHANES protocol, PFAS values below the lower limit of detection (LOD) were imputed by the CDC using standard substitution methods (LOD/√2). The proportion of values below the LOD was low, and imputation did not meaningfully influence the distribution of PFAS concentrations [17].

The study also incorporated social and behavioral factors to examine how they may influence or interact with the effects of PFAS on liver health. These factors included socioeconomic information (income, education) and lifestyle factors (smoking and alcohol consumption). These factors are known to significantly affect liver function and disease progression, making them essential for exploring their potential roles in modulating the effects of PFAS exposure.

#### 2.1.2. Blood Measurement Sample

NHANES collected biological specimens at Mobile Examination Centers (MECs) to perform laboratory analyses that offer insight into participants’ health and nutritional status. Blood samples were obtained based on participants’ age, with a minimum volume of 0.5 mL of serum. The specimens were stored in polypropylene or polyethylene containers. After collection, the blood was processed and kept refrigerated or frozen before being shipped to certified laboratories across the United States [18]. NHANES collected blood samples between 2017 and 2018 at MECs. Following collection, the samples were processed and stored at temperatures ranging from −20 °C to −70 °C, in accordance with CDC’s standardized protocols, until PFAS analysis was conducted at certified laboratories [16].

### 2.2. PFAS Extraction

To extract and quantify PFAS from human serum, tandem mass spectrometry, chromatography, and dilution were employed. The method produced accurate data on PFAS exposure by enabling the sensitive detection and quantification of PFAS in human serum with a low detection limit in the few parts per billion range.

All PFAS analytes in the dataset had a constant detection limit. Moreover, “0” values denoted outcomes that were at or above the detection limit, and “1” values indicated results that were below it. For values below the lower limit of detection, an imputed fill value was calculated. The detection limits for both PFAS and PFOS were 0.10 ng/mL [18].

### 2.3. Liver Markers

In our investigation, we utilized data derived from NHANES, which the CDC analyzed by the standardized protocols delineated in the *NHANES Laboratory Procedures Manual* to evaluate critical biomarkers of hepatic function. ALT activity was quantified via a kinetic enzymatic assay that monitors the oxidation of NADH to NAD at 340 nm, with the rate of conversion being directly proportional to ALT activity in serum. AST was determined using a modified International Federation of Clinical Chemistry (IFCC) method, which similarly measures NADH oxidation at 340 nm as an indicator of enzymatic activity. ALP concentrations were determined using a colorimetric assay that involves the enzymatic hydrolysis of *p*-nitrophenol phosphate, resulting in a chromogenic product detectable at 450 nm. GGT activity was evaluated using a kinetic rate-based assay, wherein the formation of a colored compound is observed at 415 nm. Total bilirubin concentrations were determined photometrically at 546 nm following the generation of a red azo dye complex in an acidic medium. These biomarkers were selected for their established relevance in assessing hepatocellular integrity and overall liver function. The CDC adhered to NHANES’ rigorously standardized methodologies to ensure the reliability and reproducibility of results across the study population.

#### Fatty Liver Index for Metabolic Dysfunction-Associated Steatotic Liver Disease (MASLD)

The fatty liver index (FLI) was utilized for MASLD prediction due to the absence of abdominal ultrasound results in the dataset. It has been validated as a reliable predictor of NAFLD within the United States. US-FLI is computed through a logistic regression model, incorporating Body Mass Index (BMI), waist circumference, GGT, and triglycerides (TG), as depicted in the equation below. In our study, the US FLI was calculated as follows:

$$FLI=\frac{\exp\left(A\right)}{1+\exp\left(A\right)}\;\times \;100$$

where A = 0.953 ∗ log (TG) + 0.139 ∗ BMI + 0.718 ∗ log (GGT) + 0.053 ∗ waist circumference-15.745, and log(.) is the natural logarithm.

### 2.4. Assessment of Alcohol Use and Smoking Behavior

Alcohol consumption was evaluated using data from the NHANES Alcohol Use Questionnaire, which collects information on both lifetime and current drinking habits. Participants self-reported their frequency of alcohol intake over the previous year, choosing from 11 response options ranging from “Every day” to “Never in the last year.” This detailed categorization allowed for the identification of various drinking patterns, from daily or near-daily consumption to occasional or no use, facilitating a more refined analysis of alcohol use in relation to other study variables.

Smoking behavior was assessed through responses to the NHANES Smoking—Cigarette Use questionnaire, which asks participants to report the average number of cigarettes smoked per day over the past 30 days. Reported values ranged from 0 to 60 cigarettes daily, capturing the full spectrum from non-smokers to heavy smokers. This measure provided a detailed profile of smoking intensity, supporting the examination of its associations with other health-related factors in the study.

### 2.5. Statistical Analysis

Descriptive statistics, including means and standard deviations for continuous variables and frequencies for categorical variables, were computed. Linear regression models were employed to investigate the relationship between demographic factors and liver injury biomarkers.

This study used complete data for PFAS and liver markers. Other missing values in the survey were imputed using the Amelia technique, a multiple imputation method based on an expectation–maximization with a bootstrapping algorithm that assumes data are missing at random (MAR). Amelia generates multiple complete datasets by modeling the joint distribution of the variables, allowing for valid statistical inference that incorporates uncertainty from missing data.

BKMR was employed to evaluate the combined effects of PFAS exposure, social, and behavioral factors on liver health. This approach enables the assessment of potential nonlinear relationships and interactions among exposures. BKMR estimates the model $Y=h\left(Z\right)+X\beta +\epsilon Y=h\left(Z\right)+X\beta +\epsilon$, where Y represents the health outcome, Z is the matrix of exposures, X is a matrix of covariates, β is a vector of regression coefficients, and ϵ is the error term, addressing uncertainties related to the estimation of a high-dimensional set of exposures and multiple-testing corrections [19].

Spearman’s rank correlation was performed to assess the associations between PFAS exposure, social and behavioral factors, and liver health indicators. The equation for Spearman’s rank correlation is $P\;=\;1\;-\;\frac{6\Sigma {\mathrm{d}}_{i}^{2}}{⋂\left(n^{2}\;-\;1\right)}$, where ${\mathrm{d}}_{\dot{i}}=\;$difference between the ranks of two variables and *n* = sample size

Multiple linear regression (MLR) models were used to evaluate associations between PFAS exposure, behavioral factors (such as alcohol and smoking), socioeconomic factors (income and education), and liver biomarkers. Predictor selection was based on theory and prior research, with all models adjusted for age, sex, and race/ethnicity. Statistical significance was determined at a *p*-value threshold of <0.05.

## 3. Results

### 3.1. Descriptive Statistics Results

Table 1 below represents the demographic, behavioral, and clinical characteristics of the study sample (N = 1784). The original NHANES 2017–2018 dataset included 9254 participants. However, our analytic sample was limited to individuals who had complete data for key variables required in the study. Specifically, participants were included only if they had measured serum levels of both PFOA and PFOS, complete liver function biomarker data (ALT, AST, ALP, GGT, total bilirubin, and US-FLI), and available information on relevant social and behavioral factors such as income, education, alcohol use, and smoking status. After applying these inclusion criteria and excluding participants with missing data, the final sample size was reduced to 1784. The average age of participants was 45.27 years (SD = 20.85), indicating a wide age range. The mean concentrations of PFAS chemicals were 1.71 ng/mL for PFOA and 6.51 ng/mL for PFOS, showing measurable environmental exposure across the sample. Liver injury markers, including AST, ALT, ALP, and GGT, were also reported, with ALT and AST showing similar average levels (~21 U/L), and ALP averaging around 88 U/L. The mean GGT was 24.48 U/L, and total bilirubin averaged 0.46 mg/dL, suggesting essentially normal liver function overall. The mean US-FLI was 49.14, indicating a mixed-risk population for fatty liver disease. Gender was almost evenly distributed (50.3% female, 49.3% male).

The distribution of alcohol consumption and smoking among study participants is found in Table 2.

### 3.2. Spearman Correlation Results

**Figure 1:**
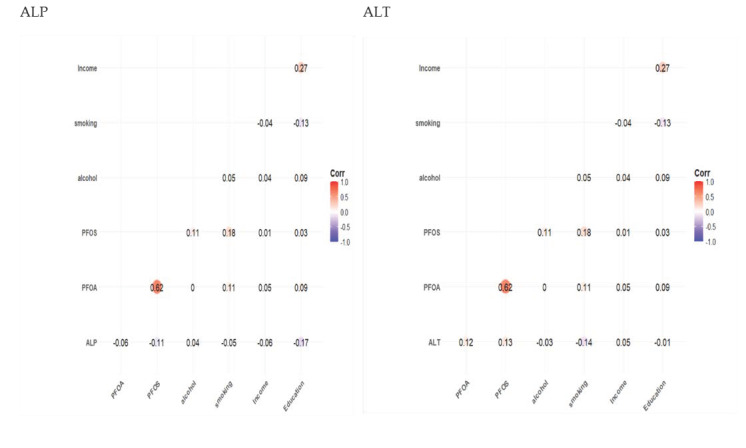

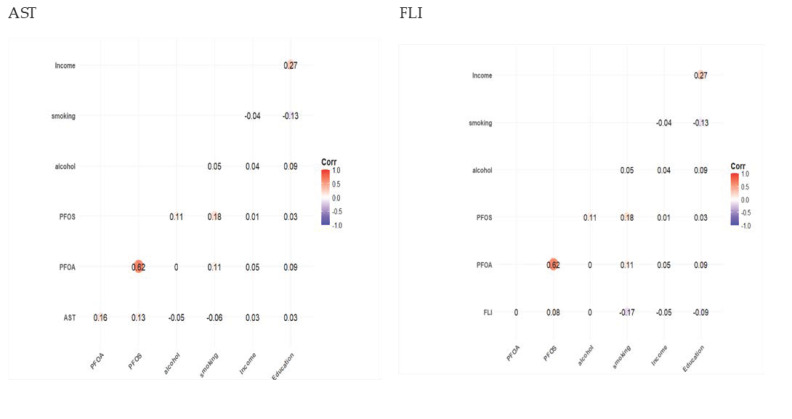

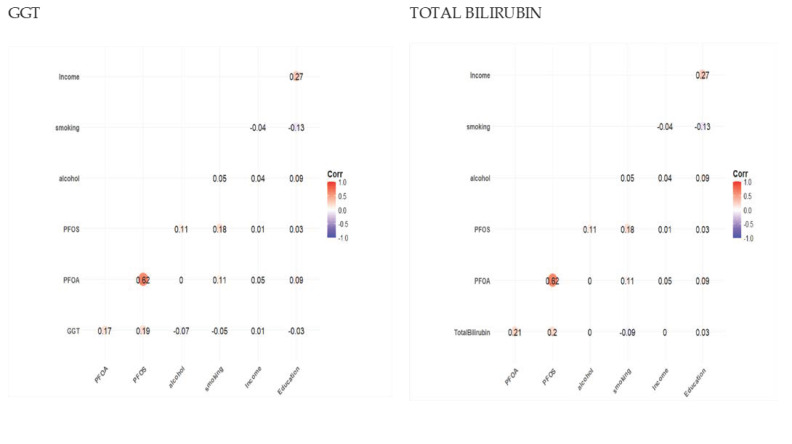
Spearman correlation matrix of key predictor variables and liver biomarkers of interest. Correlation coefficients reflect monotonic associations between variables without assuming linearity or normality.

Figure 1 displays the Spearman correlation matrix showing the relationships between key exposure variables (PFOA, PFOS, alcohol consumption, smoking, income, and education) and liver health biomarkers (ALT, AST, ALP, GGT, total bilirubin, and US-FLI). Color intensity indicates the strength and direction of each correlation, with blue representing positive correlations and red indicating negative correlations. Across markers, several trends emerged. A significant (*p* < 0.05) positive correlation between PFOA and PFOS was found across liver markers. PFOS tended to have significant, though weaker, positive associations with alcohol use and smoking, and other PFAS markers showed similar patterns. Neither PFAS marker was significantly linked to the fatty liver index. The FLI was significantly (*p* < 0.05) higher in smokers and those with lower education. Alcohol consumption increased significantly with educational attainment (*p* < 0.05), while income was only significantly (*p* < 0.05) associated with education and showed no significant relationship with PFAS levels, FLI, or smoking.

### 3.3. Linear Regression Results

Table 3 below displays linear regression results assessing the relationships between environmental contaminants (PFOA, PFOS) and socio-behavioral variables (alcohol use, smoking, income, and education) with liver injury markers (AST, ALT, ALP, GGT, total bilirubin, and FLI). The models were adjusted for age, race, and sex.

### 3.4. Bayesian Kernel Machine Regression Results

BKMR is a statistical modeling approach that enabled the study to examine the joint effects of multiple exposures of PFAS and social and behavioral factors on health outcomes. It was useful for capturing nonlinear relationships and interactions between variables that traditional linear regression models might miss.

#### 3.4.1. Posterior Inclusion Probability (PIP)

The PIP was derived from the BKMR model to evaluate the relative importance of each exposure variable in explaining variability in liver health outcomes. PIPs reflect the posterior probability that a given variable is included in the model, conditional on the data and other covariates. Rather than representing significance or relying on fixed thresholds, PIPs provide a probabilistic measure of variable relevance across the ensemble of models sampled from the posterior distribution [19]. Table 4 summarizes the PIP values for liver injury markers (ALT, AST, ALP, GGT, FLI, and total bilirubin). Group PIP represents the probability that a group of exposures (e.g., PFAS, behavioral factors) contributes to the health outcome, while conditional PIP reflects the probability that a specific exposure within that group is important, given the group is included in the model.

#### 3.4.2. Univariate Dose Responses

**Figure 2:**
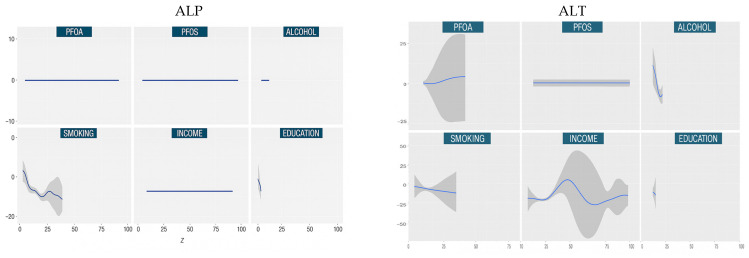

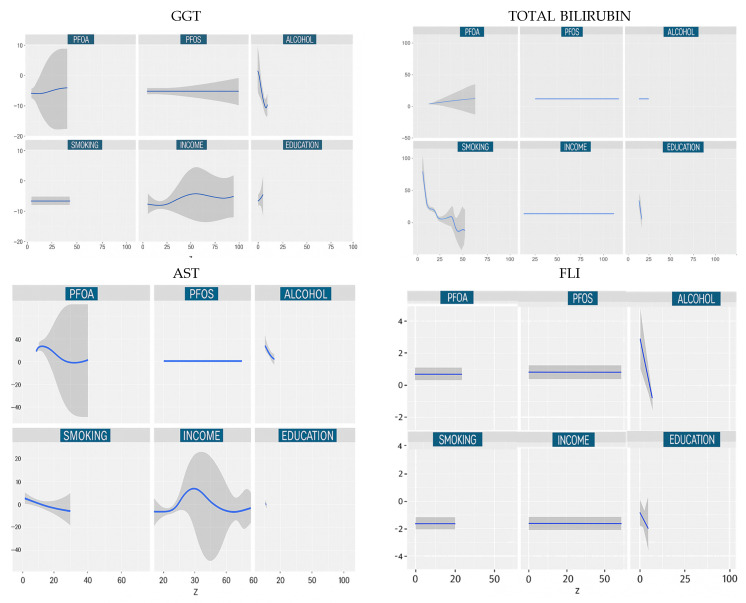
Univariate exposure–response and 95-percent credible interval (gray area) for each predictor variable when all other predictor variables are fixed at the 50th percentile. Adjusted for age, race, and sex.

The univariate dose–response analysis (Figure 2) serves to elucidate the marginal relationship between each individual exposure, such as PFOA, PFOS, alcohol consumption, smoking, income, or education, and specific liver health outcomes. This analysis is conducted by systematically varying one exposure across its observed concentration or value range, while holding all other exposures and covariates constant at a reference level, in this case, the median. This controlled framework enables the estimation of the conditional, potentially nonlinear effect of each predictor, effectively isolating its contribution within the broader exposure mixture.

The resulting exposure–response curves offer detailed insights into the shape and magnitude of each association. In our study, these curves revealed a range of patterns, including U-shaped, M-shaped, and monotonic trends, across liver biomarkers such as ALT, AST, ALP, and GGT. These flexible, data-driven relationships would likely be obscured using traditional linear models and underscore the advantage of BKMR in environmental epidemiology, particularly when exposures are correlated or exhibit complex interactions. The univariate analysis thus provided a critical lens through which to identify which exposures exert the strongest independent influence on liver function in the presence of co-occurring behavioral and environmental stressors.

#### 3.4.3. Bivariate Exposure Response

**Figure 3:**
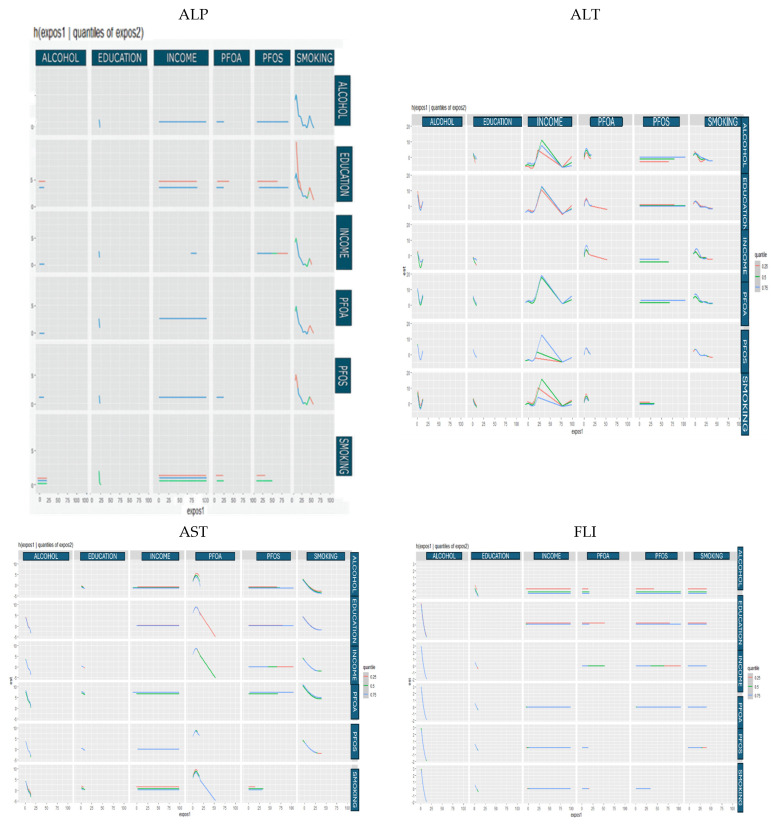

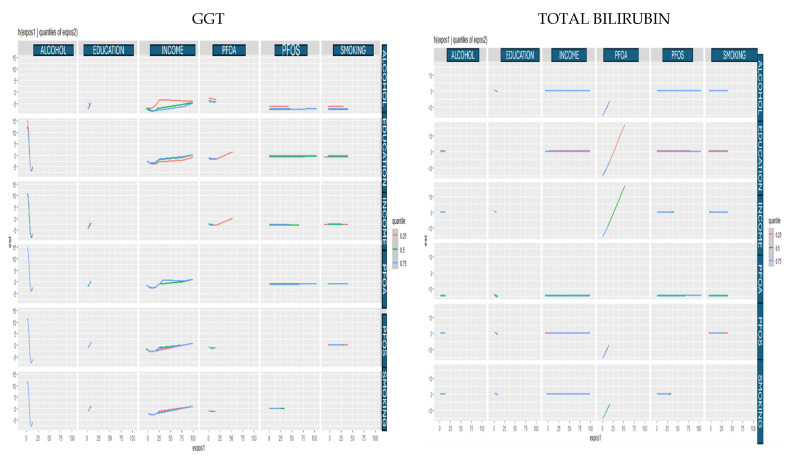
Bivariate exposure–response for each predictor variable with the first predictor variable (*x*-axis) increasing from left to right and the second predictor variable fixed at the 0.25, 0.50, and 0.75 quantiles while all other exposures are fixed at the 50th percentile. Adjusted for age, race, and sex.

The bivariate exposure–response analysis (Figure 3) illustrates how pairs of exposures, such as PFAS compounds and behavioral or socioeconomic factors, jointly influence liver health outcomes. In this analysis, one exposure is varied across its observed range while the second is fixed at selected quantiles (e.g., 25th, 50th, and 75th percentiles), and all remaining exposures and covariates are held constant at their median values. This allows for the visualization of interaction patterns between two exposures, providing insight into whether their combined effects on liver biomarkers are additive, synergistic, antagonistic, or nonmonotonic.

By modeling these joint effects within the BKMR framework, the analysis captures nuanced exposure–outcome relationships that may be overlooked in traditional regression models. In the context of our study, these bivariate surfaces revealed how social and behavioral modifiers, such as smoking or income, may amplify or attenuate the effects of PFAS on liver function, highlighting potential vulnerability pathways and population-level heterogeneity in risk.

#### 3.4.4. Single-Variable Risk Summary

**Figure 4:**
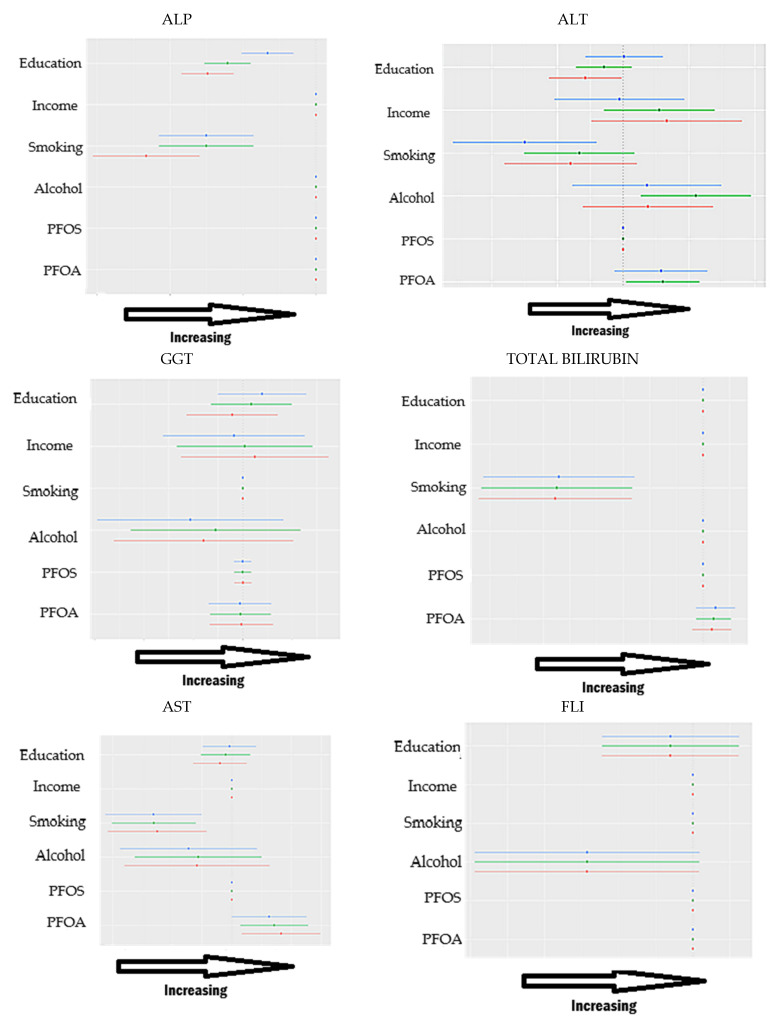
Single-exposure effect of the individual exposure on liver markers; examining the change in response associated with a change in a single exposure from its 25th to 75th quantile while all other exposures are fixed at a specific quantile (25th, 50th, and 75th). In image blue is the 0.75 quantile, green is the 0.5 quantile, and red is the 0.25 quantile. Adjusted for age, race, and sex. The *X*-axis represents the estimated change in liver dysfunction, with estimates further to the right indicating higher dysfunction.

The single-variable risk summary (Figure 4) estimates how changes in one specific exposure variable influence the liver health outcome, while holding all other exposures constant at a specified level. This approach helps researchers isolate and interpret the marginal effect of a single predictor, such as education, income, smoking, alcohol use, PFOA, or PFOS, within the context of a complex mixture of environmental and behavioral factors.

More formally, for a given predictor zj, the effect is defined as the change in the predicted liver outcome h (⋅) when zj shifts from its 25th percentile to its 75th percentile, while all other predictors z−j (the remaining exposures) are held fixed at a specific quantile, such as the 25th, 50th, or 75th percentile.

#### 3.4.5. Overall Exposure Effect

**Figure 5:**
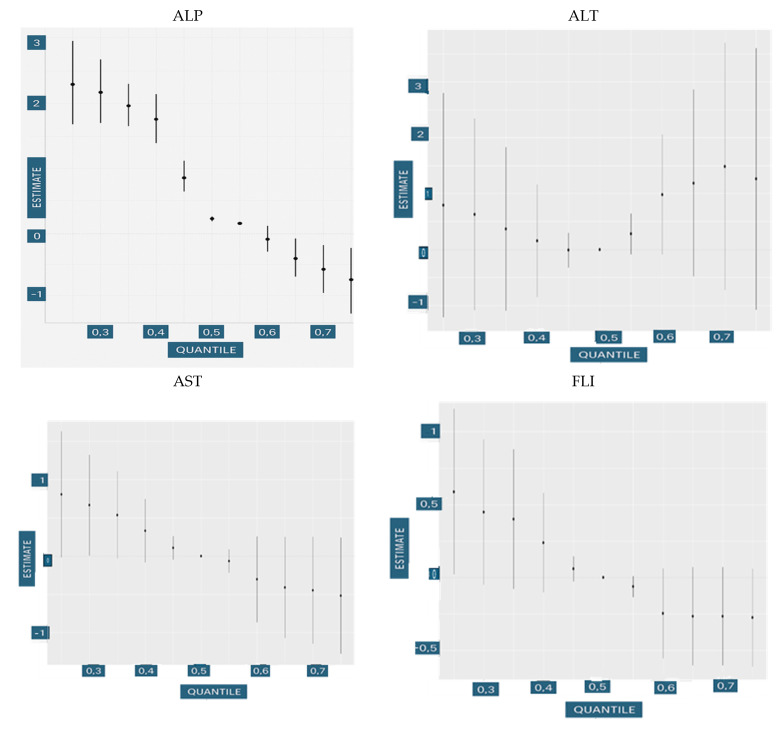

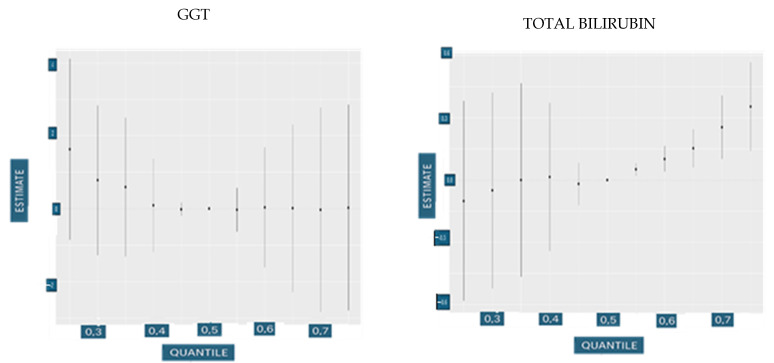
Overall exposure effect estimates for each liver biomarker. Each point represents the estimated change in the biomarker level as all exposures (PFAS, alcohol, smoking, income, and education) increase from the 25th to the 75th percentile. Adjusted for age, race, and sex. Vertical lines denote the 95% credible intervals around each estimate. Higher estimates on the *Y*-axis denote worse liver health.

The overall exposure effect analysis (Figure 5) summarizes the cumulative impact of all exposures, PFAS compounds, alcohol use, smoking, income, and education, on liver health biomarkers. Rather than isolating each exposure, this analysis estimates the predicted change in liver outcomes when all exposures are simultaneously shifted to higher percentiles (e.g., 60th, 65th, or 75th), compared to a reference scenario in which all exposures are set at their median (50th percentile), while holding covariates constant.

This approach captures the combined and potentially nonlinear influence of the full exposure mixture on liver function, providing an interpretable summary of how real-world co-exposures may affect health. The plotted estimates include 95% credible intervals, allowing for the assessment of the precision and uncertainty of the cumulative effect.

## 4. Discussion

The results indicated that behavioral factors, particularly alcohol consumption and smoking, had the strongest connections to liver biomarkers, outweighing the influence of PFAS exposure. Although the short-term effects of PFAS appeared limited in our models, their known tendency to bioaccumulate [20] suggests they could lead to long-term liver effects. In contrast, socioeconomic factors like income and education consistently emerged as strong predictors of liver health, sometimes having a greater impact than environmental exposures.

For example, linear regression analyses showed that higher income levels were generally associated with lower concentrations of liver enzymes such as AST and ALT, which are commonly elevated during liver inflammation or injury. The regression coefficients for income and AST (−0.1335; 95% CI: −0.2684 to 0.0014) and ALT (−0.1721; 95% CI: −0.3615 to 0.0172), found in Table 4, reflect a trend toward improved liver function with increased income, even if not always statistically significant.

Supporting these findings, Lee et al. [21] reported that individuals in higher income brackets had significantly lower odds of developing liver disease compared to those with lower incomes, with a 33% reduction in disease prevalence observed among higher-income groups.

Similarly, education demonstrated notable predictive value in the BKMR analysis. For ALT, education had a PIP (Table 4) of 0.5482, second only to alcohol consumption, while for GGT, education showed a group PIP of 0.3976, again outperforming PFAS. These findings suggest that higher educational attainment may protect liver health, potentially through greater health literacy, healthier behaviors, and improved access to healthcare services, even amidst environmental exposures like PFAS.

These findings are further supported when considering both the magnitude of the linear regression coefficients and the relative importance reflected by the PIPs. For instance, although PFOS had a positive coefficient of 0.54 for ALT, behavioral factors such as alcohol consumption and smoking showed similar or stronger associations (−0.29 and −0.35, respectively), along with substantially higher PIPs (0.5936 and 0.3858, respectively, compared to 0 for PFOS). Similar trends were evident for other biomarkers as well: for GGT and total bilirubin, alcohol had a PIP of 1.0 and smoking had a PIP of 0.9798, while PFOS remained negligible (e.g., 0.0014). Together, these patterns highlight the consistently stronger role of behavioral exposures in shaping liver function relative to PFAS in this population.

For example, in the linear regression models (Table 3), income was inversely associated with several liver injury markers, including AST and ALT, suggesting that individuals with higher income levels generally had lower levels of these liver enzymes. These enzymes often indicate liver inflammation or damage when elevated, so lower values can reflect better liver health. The regression coefficient for income and AST was −0.1335 (95% CI: −0.2684 to 0.0014), and for ALT it was −0.1721 (95% CI: −0.3615 to 0.0172), indicating a trend toward improved liver function with increased income, though not always statistically significant.

A study by Lee et al. [21] highlighted that individuals from higher income groups (HIGs) exhibited significantly lower odds of liver disease compared to those from lower income groups (LIGs), with adjusted odds ratios indicating a 33% lower likelihood of any liver disease in HIGs [13].

While previous studies have reported associations between PFAS exposure and liver disease [6], our findings highlight the significant role of educational attainment in influencing liver health (Table 4). In the analysis of ALT, education yielded a PIP of 0.5482, second only to alcohol consumption (PIP = 0.5936), indicating a higher likelihood of being included in models predicting ALT variability than PFAS. Similarly, for GGT, another key hepatic biomarker, education had a group PIP of 0.3976, surpassing PFAS in predictive importance.

These results suggest that education may confer protective effects against liver dysfunction, potentially through mechanisms such as increased health literacy, improved access to healthcare resources, and healthier lifestyle choices, even in the presence of environmental toxicants like PFAS.

From the BKMR univariate exposure–response analysis (Figure 2), we observed distinct and often nonlinear relationships between lifestyle, socioeconomic, and environmental predictors and the hepatic biomarkers examined. These functions, which estimate the effect of each variable while holding all others constant at their median values, revealed varying sensitivities and response profiles across biomarkers.

For ALP, only smoking and education demonstrated notable associations. Smoking exhibited a complex M-shaped pattern, suggestive of possible threshold effects or biphasic biological mechanisms, while education showed a monotonic inverse trend, consistent with protective social gradients in liver function [22,23]. PFOS, PFOA, alcohol consumption, and income displayed flat curves with narrow credible intervals, indicating negligible associations with ALP in this context.

GGT showed broader responsiveness. Alcohol consumption was associated with a steep downward trend, consistent with known metabolic interactions, though possibly reflecting complex behavioral confounding factors [24]. Income demonstrated a non-monotonic, wavy pattern, suggesting a more intricate relationship potentially shaped by socioeconomic heterogeneity in lifestyle or comorbidities.

For FLI, inverse associations were observed with both alcohol consumption and education, while other variables—including PFOS, PFOA, smoking, and income—showed no meaningful effects. This indicates more selective sensitivity, with FLI potentially capturing specific behavioral or metabolic factors rather than generalized environmental exposures [25].

AST demonstrated a smooth N-shaped relationship with PFOA, suggesting potential nonmonotonic dose–response dynamics, a hallmark of endocrine-disrupting exposures [26]. ALT exhibited multiple complex patterns: an M-shaped relationship with PFOA, a U-shaped curve with alcohol, and an inverse-U association with income. These diverse response shapes may reflect saturation effects, compensatory physiological mechanisms, or exposure-related heterogeneity [27,28].

Total bilirubin was primarily influenced by smoking, which showed a complex upward-sloping pattern. This may indicate effects mediated through oxidative stress, heme metabolism, or inflammatory processes, aligning with prior evidence linking tobacco exposure to altered bilirubin levels.

The bivariate BKMR exposure–response plots (Figure 3) offer insights into the nuanced, interactive effects of environmental, behavioral, and socioeconomic factors on hepatic biomarkers. For ALP, the flat response curves across increasing PFOA and PFOS concentrations suggest a lack of direct marginal association in this sample, diverging from prior reports of elevated ALP with PFAS exposure [29]. This discrepancy may reflect effect modification by contextual factors such as socioeconomic status or co-exposures that alter PFAS bioavailability, metabolism, or downstream signaling pathways. Alternatively, the observed flatness may indicate that ALP is less sensitive to PFAS in populations with certain protective behavioral or demographic profiles [30].

The inverse association between smoking and ALP, especially its complex, undulating pattern across exposure quantiles, may be indicative of dose-dependent liver enzyme suppression or altered hepatic enzyme induction due to chronic tobacco exposure. The presence of peaks at the 0.25 and 0.75 quantiles when smoking covaries with education and income suggests threshold effects or possible nonlinear toxicodynamics. These interactions may reflect that individuals with lower education or income experience cumulative vulnerabilities that amplify or blunt the hepatic response to tobacco exposure, perhaps via increased inflammation or stress-mediated enzyme regulation [31].

Education’s inverse relationship with ALP when interacting with other exposures reinforces the hypothesis that higher educational attainment may confer resilience to environmental insults, likely mediated through improved health behaviors, access to care, and lower chronic stress burden. That income and alcohol were largely null in their bivariate effects on ALP suggests that these factors alone may not be primary drivers of ALP variability unless interacting with more potent biological stressors.

For ALT, the V-shaped pattern associated with alcohol across other exposures may signify a biphasic hepatocellular response, where low to moderate alcohol intake initially triggers a protective or adaptive hepatic effect, but higher levels result in cytotoxic stress and enzyme leakage. This nonmonotonicity aligns with the literature, which suggests potential hormetic responses in liver enzyme regulation [32]. PFOS exhibited minimal interaction with ALT, but PFOA showed a subtle inverse-V-shaped relationship, suggesting that hepatocellular integrity may initially increase with exposure before declining, consistent with early-stage adaptive compensation followed by cellular injury.

The nonlinear ALT–smoking relationship, characterized by an early elevation and subsequent decline, may indicate changes in metabolic compensation over time. Chronic smokers may initially exhibit liver stress, but longer-term exposure could result in hepatic adaptation or suppression of transaminase activity through oxidative depletion or receptor desensitization [33,34,35].

AST shared several patterns with ALT but demonstrated more pronounced nonlinearity in its interaction with income and PFOA. The inverse-V shape for income may reflect the compounding physiological effects of economic stress, which, when moderate, may provoke hepatic stress responses but at high levels may indicate underlying selection biases (e.g., survivors or those receiving medical care). The biphasic PFOA pattern, characterized by an early rise followed by a decline, may indicate a transition from an adaptive to a pathophysiological liver state with increasing toxic burden. The persistent inverse relationship between smoking and AST suggests chronic hepatic enzyme suppression or altered mitochondrial function.

For FLI, the strong negative interaction between alcohol and other exposures, especially at the upper quantiles, underscores alcohol’s dominant role in hepatic lipid accumulation and steatosis. The heightened effect at the 0.75 quantile suggests that individuals with higher socioeconomic stressors or environmental exposures may experience exacerbated liver dysfunction when alcohol is introduced, potentially via synergistic inflammatory or endocrine-disrupting pathways. The inverse pattern for education with FLI supports its protective role, likely through behavioral and lifestyle mediation [36,37]. The null bivariate patterns for other variables indicate that FLI may be more susceptible to specific lifestyle modifiers than chemical exposures in this context.

For GGT, alcohol again displayed an inverse relationship in the joint exposure space, unexpected given alcohol’s known hepatotoxicity. This may reflect sample characteristics, underreporting, or a differential hepatic enzyme response among light or moderate drinkers. Slight increases in GGT with education and income suggest potential confounding or behavioral correlations (e.g., higher alcohol quality, access to health-promoting resources). The flat responses for PFAS and smoking further imply that GGT may not be an early or sensitive biomarker for these exposures in multivariable contexts, possibly due to saturation of oxidative stress pathways or compensatory homeostatic mechanisms.

Finally, the most prominent bivariate finding for total bilirubin involved PFOA, with a consistent monotonic increase across other exposures. Elevated bilirubin in this context may signal oxidative stress or mild hepatocellular dysfunction, potentially through PFOA-induced interference with bilirubin conjugation and excretion. Given bilirubin’s dual role as both a marker of liver injury and an endogenous antioxidant, this elevation may reflect compensatory upregulation in response to PFAS-induced stress rather than direct hepatocellular damage [38].

The single-variable and interaction effect analysis (Figure 4 and Figure 5) from the BKMR model quantified how individual exposures influenced hepatic biomarkers both independently and within the context of the broader exposure mixture. Specifically, we assessed how moving one exposure from its 25th to 75th percentile affected biomarker levels under different fixed quantile conditions of the remaining variables. The interaction component extended this by examining how the effect of a single exposure shifted when all other exposures were simultaneously varied in the opposite direction (from 75th to 25th percentile), capturing the extent to which an individual exposure’s influence was modified by the presence of the remaining mixture.

For ALP, education and smoking emerged as the most prominent variables showing interaction effects with other exposures. This indicates that ALP levels are particularly sensitive to the social and behavioral environment. The inverse association with smoking, modulated by varying levels of other exposures, may reflect hepatic enzyme suppression due to chronic oxidative stress. Meanwhile, education’s consistent interaction pattern suggests it plays a contextual role, potentially buffering the hepatotoxic effects of exposures through behavioral modification or enhanced access to preventive health resources.

In the case of ALT, education, income, smoking, and alcohol all demonstrated interaction effects with nearly all other exposures in the mixture. This implies a high degree of contextual sensitivity in ALT responses. ALT, a key indicator of hepatocellular injury, appears to integrate signals not only from direct chemical insults but also from broader lifestyle and socioeconomic stressors. These findings suggest a biologically plausible pathway in which environmental exposures interact with systemic stress, alcohol metabolism, and health behavior to influence liver enzyme levels.

For GGT, education, income, and alcohol showed consistent interactions with other variables. GGT reflects both hepatobiliary function and systemic oxidative stress. The interaction effects suggest that GGT may serve as a sensitive integrative marker of environmental and behavioral stressors, particularly when individuals face co-occurring exposures such as alcohol use in the context of differing socioeconomic status. The slight increases associated with higher income and education may reflect health behavior differences or unmeasured covariates such as diet or access to screening, which could influence enzyme induction patterns.

In contrast, total bilirubin did not exhibit significant interaction effects across any exposure combination. This suggests a relative independence from environmental-behavioral co-exposures, potentially due to bilirubin’s role as an antioxidant and its regulation through well-conserved metabolic pathways [39]. Alternatively, its lack of sensitivity to interaction may result from genetic determinants dominating its variability in this population, limiting its utility as a marker of interactive exposure effects.

AST showed the strongest interaction effects for education and PFOA across other exposures. AST responds to a range of hepatic and mitochondrial stressors, and its sensitivity to PFOA may reflect the ability of PFAS to disrupt mitochondrial energy metabolism or promote low-grade hepatic inflammation. Education’s interaction suggests that structural determinants of health influence not only the baseline risk but also potentially modulate physiological responses to environmental toxicants.

Finally, for FLI, there was limited evidence of interaction effects across all variables. FLI, being a composite index incorporating metabolic indicators, may be less responsive to short-term fluctuations in individual exposures and more indicative of long-term cumulative risk. The lack of strong interactions could suggest that FLI is primarily driven by dominant contributors such as alcohol intake or adiposity, which may overshadow more subtle interactive influences of environmental exposures in this modeling context.

To evaluate the joint effect of the entire exposure mixture on liver-related biomarkers, we employed the overall risk summary functionality available within the BKMR framework. Specifically, this analysis estimates the predicted change in the outcome as all exposure variables are simultaneously set to increasing quantiles of their empirical distributions, ranging here from the 25th to the 75th percentile, while holding all covariates fixed and using the median exposure level as the reference point. This approach generates an interpretable measure of the cumulative impact of the full mixture, reflecting potential nonlinearities and interactions inherent in the joint exposure–response surface. It does not assume additivity or linearity and is particularly well suited for high-dimensional, correlated exposures.

For AST and FLI, we observed a consistent negative trend in response to increasing mixture quantiles. This indicates that higher joint exposure to the mixture was associated with reduced levels of these biomarkers. One possible interpretation is a nonlinear suppression effect, where certain pollutants exert biologically dampening influences on AST and FLI at higher doses. Alternatively, the effect may reflect the dominance of exposures with inverse associations in the mixture. Because BKMR models the joint effect without imposing parametric constraints, the overall risk summary is shaped by the integrated influence of all individual exposure–response relationships and their covariance structure. The relatively narrow credible intervals accompanying these trends suggest posterior stability and greater model certainty in the estimated effects.

ALP also showed a downward trend across the quantile range, with particularly narrow 95% credible intervals. This implies a more precise and consistent inverse association across posterior samples. The greater precision likely reflects low variance in the estimated exposure–response functions, which can occur when several exposures with consistent monotonic effects dominate the kernel-weighted signal [19]. In such cases, the statistical evidence for a joint association is both strong and stable, reinforcing the reliability of the observed negative relationship.

For ALT and GGT, the overall mixture effect revealed U-shaped relationships, with both lower and higher quantiles associated with elevated biomarker levels relative to the median. These nonmonotonic dose–response patterns are consistent with hypothesized mechanisms for endocrine-disrupting compounds or hormetic responses, where mid-range exposures suppress biomarker activity [40], while low or high exposures may stimulate compensatory physiological pathways. BKMR’s kernel-based estimation enables such nonmonotonic effects to emerge organically from the data. However, the credible intervals for these biomarkers, particularly GGT, were wider, indicating greater posterior uncertainty. This may stem from weaker or less consistent associations across individual exposures, higher variability in the outcome, or challenges in identifying stable joint effects under conditions of multicollinearity.

In contrast, total bilirubin exhibited a positive overall trend, with increasing mixture quantiles corresponding to elevated biomarker levels. This relationship may be driven by pollutants with positive marginal effects on bilirubin or by synergistic interactions among exposures that elevate hepatic stress or oxidative burden. Given BKMR’s ability to jointly condition on correlated exposures, this observed trend likely reflects the dominant direction of the combined influence across the mixture, rather than any single component.

Taken together, these results highlight the capacity of BKMR to flexibly estimate joint mixture effects while accounting for complex exposure dependencies, nonlinear relationships, and model uncertainty. The heterogeneity in overall response shapes across biomarkers underscores the importance of data-adaptive modeling approaches when evaluating environmental mixtures with potentially diverse modes of action.

Although our models did not provide strong or consistent evidence that PFAS significantly worsen liver dysfunction when combined with behavioral or socioeconomic risk factors, the possibility of complex, nonlinear interactions remains. The BKMR analysis revealed modest interaction patterns between PFAS and factors such as education and income, indicating that vulnerability to PFAS-related liver effects may differ across population groups. These findings highlight the need for future research to more deeply examine how environmental exposures interact with social and behavioral determinants to influence liver health outcomes.

### Limitations

This study has several limitations that should be considered. First, the cross-sectional design of NHANES prevents causal conclusions; the observed links between PFAS exposure, behavioral and social factors, and liver biomarkers are correlations, not evidence of cause and effect. Exposures or factors were assessed at a single point in time and might not accurately represent long-term or accumulated exposure, especially for persistent chemicals like PFAS. Third, relying on self-reported behavioral data (such as alcohol use and smoking) may lead to misclassification or recall bias. Additionally, unmeasured variables like diet, occupational exposure, or genetic predisposition could confound the results. Finally, while BKMR offers a flexible way to model complex exposure mixtures, it depends on several assumptions, including a proper kernel choice and the convergence of the Bayesian algorithm.

Another limitation of this study is its reliance on pre-pandemic data from the 2017–2018 NHANES cycle. Although this dataset offers robust information on PFAS exposure and liver biomarkers, it does not reflect potential shifts in environmental exposures, health behaviors, or socioeconomic conditions that occurred during and after the COVID-19 pandemic. As such, future research incorporating post-pandemic data will be important to determine whether the associations observed here remain consistent or have changed in response to pandemic-related influences.

These limitations indicate that findings should be approached with caution. Specifically, the policy implications of this study—such as focusing on behavioral interventions for liver health—should be viewed as preliminary. Further longitudinal studies and controlled experiments are necessary to determine causality and assess how changes in PFAS exposure or behavioral risk factors affect liver outcomes over time.

## 5. Conclusions

Our findings suggest that lifestyle and socioeconomic factors may exert stronger short-term effects on liver biomarkers than PFAS exposure. Behaviors such as drinking alcohol and smoking have the strongest links to liver problems. Social factors (income and education) also play a major role in liver health. People with higher incomes and higher levels of education generally had better liver health.

From our study, it is important to note that improving liver health in a meaningful way requires more than just controlling chemical exposures; it also means addressing social and behavioral factors. While PFAS alone showed weaker associations, our mixture models identified modest interactions, particularly with income and education, highlighting the need for further research to assess whether PFAS may contribute to liver dysfunction in the presence of other stressors.

## Figures and Tables

**Table 1:** Characteristics of the sample participants.

Characteristics	Total Sample Population N = 1784
Demographic	
Age: mean (SD)	45.27 (20.85)
Gender—female	50.3%
Gender—male	49.3%
PFAS	
PFOA: mean (SD)	1.71 (1.82)
PFOS: mean (SD)	6.51 (7.74)
Liver Markers	
AST: mean (SD)	21.26 (10.64)
ALT: mean (SD)	21.27 (16.27)
ALP: mean (SD)	87.97 (46.81)
GGT: mean (SD)	28.48 (35.19)
Total bilirubin: mean (SD)	0.46 (0.29)
US-FLI: mean (SD)	49.14 (33.60)
Gender—female	50.3%
Gender—male	49.3%
Education	
Education—less than 9th grade	9.0%
Education—9–11th grade (includes 12th grade with no diploma)	11.4%
Education—high school graduate/GED or equivalent	22.9%
Education—some college or AA degree	33.3%
Education—college graduate or above	23.4%
Race/Ethnicity	
Race/ethnicity—Black	23.4%
Race/ethnicity—White	36.6%
Mexican American	16.2%
Race/ethnicity—other Hispanic	9.7%
Race/ethnicity—Asian	14.1%
Income	
USD 0 to USD 4999	2.79%
USD 5000 to USD 9999	2.97%
USD 10,000 to USD 14,999	5.75%
USD 15,000 to USD 19,999	6.48%
USD 20,000 to USD 24,999	7.57%
USD 25,000 to USD 34,999	11.27%
USD 35,000 to USD 44,999	12.48%
USD 45,000 to USD 54,999	9.21%
USD 55,000 to USD 64,999	5.81%
USD 65,000 to USD 74,999	5.94%
USD 75,000 to USD 99,999	10.66%
USD 100,000 and over	19.08%

**Table 2:** Distribution of alcohol consumption and smoking behavior categories.

Behavior	Category	% of Participants
Alcohol use (number of days with 4 or 5 drinks in the past 12 months)	Every day	0.81
	Nearly every day	0.9
	3 to 4 times a week	1.44
	2 times a week	2.97
	Once a week	3.24
	2 to 3 times a month	6.76
	Once a month	4.32
	7 to 11 times in the last year	2.34
	3 to 6 times in the last year	6.4
	1 to 2 times in the last year	11.71
	Never drank in past year	59.1
Smoking (average number of cigarettes per day in the last 30 days)	1–5	32.30
	6–10	34.36
	11–15	7.90
	16–20	20.27
	21–25	1.03
	26–30	2.75
	31–35	0.34
	36–40	1.03

**Table 3:** Linear regression results for PFAS, lifestyle, and social factors with liver-related variables.

Outcome	Variable	* Coef. Estimate	Std Deviation	*t*-Value	95% CI Lower	95% CI Upper
AST	PFOA	0.1472	1.0602	0.1388	−1.9441	2.2385
	PFOS	0.0959	0.2234	0.4292	−0.3447	0.5365
	Alcohol	−0.1062	0.3771	−0.2815	−0.8501	0.6377
	Smoking	−0.2379	0.1602	−1.4854	−0.5538	0.0780
	Income	−0.1335	0.0684	−1.9527	−0.2684	0.0014
	Education	0.6615	1.1664	0.5672	−1.6391	2.9621
ALT	PFOA	−1.4313	1.4867	−0.9627	−4.3636	1.5011
	PFOS	0.5429	0.3134	1.7320	−0.0754	1.1611
	Alcohol	−0.2909	0.5296	−0.5492	−1.3353	0.7537
	Smoking	−0.3477	0.2241	−1.5517	−0.7896	0.0943
	Income	−0.1721	0.0960	−1.7927	−0.3615	0.0172
	Education	0.5660	1.6383	0.3454	−2.6655	3.7974
ALP	PFOA	2.5916	1.4739	1.7584	−0.3154	5.4987
	PFOS	0.2012	0.3107	0.6474	−0.4117	0.8141
	Alcohol	1.0012	0.5250	1.9070	−0.0343	2.0366
	Smoking	−0.0267	0.2221	−0.1203	−0.4648	0.4113
	Income	−0.0323	0.0952	−0.3391	−0.2200	0.1555
	Education	−0.9675	1.6242	−0.5957	−4.1710	2.2361
GGT	PFOA	−1.8948	3.5798	−0.5293	−8.9556	5.1660
	PFOS	2.0973	0.7548	2.7788	0.6086	3.5860
	Alcohol	−1.9580	1.2751	−1.5355	−4.4730	0.5571
	Smoking	−0.4319	0.5395	−0.8005	−1.4960	0.6322
	Income	−0.4221	0.2312	−1.8258	−0.8781	0.0339
	Education	0.1770	3.9450	0.0449	−7.6040	7.9581
Total Bilirubin	PFOA	0.0086	0.0153	0.5602	−0.0216	0.0388
	PFOS	0.0014	0.0032	0.4191	−0.0050	0.0077
	Alcohol	−0.0060	0.0055	−1.0954	−0.0167	0.0048
	Smoking	−0.0040	0.0023	−1.7543	−0.0086	0.0005
	Income	−0.0005	0.0010	−0.5551	−0.0025	0.0014
	Education	0.0172	0.0169	1.0190	−0.0161	0.0504
US FLI	PFOA	−1.9104	3.4856	−0.548	−8.8360	5.0151
	PFOS	0.7668	0.6638	1.155	−0.5506	2.0842
	Alcohol	−0.6863	1.1666	−0.588	−2.0039	3.3762
	Smoking	−0.9560	0.5334	−1.792	−2.0151	0.1031
	Income	−0.2706	0.1778	−1.522	−0.6244	0.0832
	Education	4.3534	3.8554	1.129	−3.3115	12.0183

* Adjusted for age, race, and sex.

**Table 4:** Posterior Inclusion Probability (PIP) results.

PIP VALUES FOR ALT				
	EXPOSURE	Group	Group PIP	Conditional PIP
PFOA	PFOA	1	0.3312	0.3312
PFOS	PFOS	1	0.3312	0.0000
Alcohol	Alcohol	2	0.9794	0.5936
Smoking	Smoking	2	0.9794	0.3858
Income	Income	3	0.6676	0.1194
Education	Education	3	0.6676	0.5482
PIP VALUES FOR ALP				
	EXPOSURE	Group	Group PIP	Conditional PIP
PFOA	PFOA	1	0.001	0.0002
PFOS	PFOS	1	0.001	0.0008
Alcohol	Alcohol	2	1.000	0.0016
Smoking	Smoking	2	1.000	0.9984
Income	Income	3	1.000	0.0004
Education	Education	3	1.000	0.9996
PIP VALUES FOR AST				
	EXPOSURE	Group	Group PIP	Conditional PIP
PFOA	PFOA	1	0.0480	0.0472
PFOS	PFOS	1	0.0480	0.0008
Alcohol	Alcohol	2	0.8878	0.8628
Smoking	Smoking	2	0.8878	0.0250
Income	Income	3	0.0192	0.0004
Education	Education	3	0.0192	0.0188
PIP VALUES FOR FLI				
	EXPOSURE	Group	Group PIP	Conditional PIP
PFOA	PFOA	1	0.0024	0.0004
PFOS	PFOS	1	0.0024	0.0020
Alcohol	Alcohol	2	0.0426	0.0416
Smoking	Smoking	2	0.0426	0.0010
Income	Income	3	0.0056	0.0004
Education	Education	3	0.0056	0.0052
PIP VALUES FOR GGT				
	EXPOSURE	Group	Group PIP	Conditional PIP
PFOA	PFOA	1	0.1298	0.1290
PFOS	PFOS	1	0.1298	0.0008
Alcohol	Alcohol	2	1.0000	1.0000
Smoking	Smoking	2	1.0000	0.0000
Income	Income	3	0.3976	0.0316
Education	Education	3	0.3976	0.3660
PIP VALUES FOR TOTAL BILIRUBIN				
	EXPOSURE	Group	Group PIP	Conditional PIP
PFOA	PFOA	1	0.0236	0.0222
PFOS	PFOS	1	0.0236	0.0014
Alcohol	Alcohol	2	0.9814	0.0016
Smoking	Smoking	2	0.9814	0.9798
Income	Income	3	0.0002	0.0002
Education	Education	3	0.0002	0.0000

## Data Availability

The NHANES dataset is publicly available online, accessible at https://www.cdc.gov/nchs/nhanes/index.html (accessed on 1 May 2025).
